# κ-Carrageenan and Its Synergistic Blends: Next-Generation Food Gels

**DOI:** 10.3390/gels11120976

**Published:** 2025-12-04

**Authors:** Simona Russo Spena, Nino Grizzuti

**Affiliations:** Dipartimento di Ingegneria Chimica, Dei Materiali e Della Produzione Industriale, Università degli Studi di Napoli Federico II, P.le Tecchio 80, 80125 Naples, Italy; simona.russospena@unina.it

**Keywords:** plant-based hydrocolloids, gelatin alternatives, κ-carrageenan, synergistic gels, food application

## Abstract

The growing demand for plant-based foods, together with environmental and ethical concerns associated with animal-derived ingredients, has intensified research into alternative gelling agents for the food industry. Within this framework, the review focuses on the use of vegetable hydrocolloids in dairy analogues, confectionery-type gels, and emerging 3D-printed food systems. Plant-based hydrocolloids have emerged as promising candidates to replace animal gelatin across diverse applications. This review highlights recent advances in the use of plant-based hydrocolloids, focusing on κ-carrageenan (κ-C) and its blends with other plant-based gums as functional gelling systems for food products. Particular attention is given to synergistic combinations of κ-C with other hydrocolloids (e.g., locust bean gum, konjac glucomannan, and starches) as strategies to modulate gel strength, stability, and sensory properties. The mechanical and sensory performance of these systems is critically examined. Key advantages of plant hydrocolloids are discussed, such as their versatility, compatibility with a range of ingredients, and gelation under varied conditions, along with their limitations, including difficulties in replicating gelatin’s sensory profile, formulation challenges, and sensitivity to processing parameters. Finally, the review identifies future research directions and formulation strategies aimed at developing innovative plant-based gels that meet both the technological and sensory expectations of manufacturers and consumers in the food sector.

## 1. Introduction

The steady increase in the global population is accompanied by a growing demand for food resources. Food production currently accounts for an estimated 20–30% of the total environmental impact generated by human activities [1]. Among the various sectors, animal-derived foods such as meat and dairy are recognized as major contributors to greenhouse gas emissions, biodiversity loss, and animal welfare concerns. In response, consumers are showing greater awareness of sustainability issues, including environmental protection and ethical considerations, which has led to a marked rise in vegetarian and vegan diets [2]. Within this context, the development of alternatives to animal-derived ingredients has become an urgent priority.

One of the most critical challenges in this transition involves food gels, which play a central role in imparting texture and functionality to a wide range of products. Conventional gels are largely based on animal-derived gelatin, making the identification of sustainable, plant-based substitutes a key research focus. Food gels are a fundamental class of structured materials in modern food systems, providing stability, texture, and desirable sensory experiences. They are employed across diverse applications, including confectionery, dairy and plant-based alternatives, desserts, meat analogues, and more recently in emerging fields such as 3D-printed foods. The ability of gels to integrate mechanical performance with consumer-perceived attributes makes them indispensable in food design. Nevertheless, most traditional systems still rely heavily on animal gelatin.

Plant-based hydrocolloids represent one of the most promising routes for designing next-generation food gels. Sourced from renewable plant materials such as seaweeds, seeds, and legumes, they provide sustainable and versatile alternatives to animal gelatin. Although they may not fully replicate all of gelatin’s unique properties, vegetable hydrocolloids offer excellent gelling, thickening, and stabilizing abilities. Moreover, their functionality can be enhanced through strategic combinations, which help to overcome individual limitations. In particular, κ-carrageenan (κ-c) and its synergistic blends with other plant-based gums (e.g., locust bean gum, konjac, starches) have emerged as powerful systems for tailoring gel strength, stability, and sensory perception.

Several review articles [3,4,5,6,7] have discussed the structure and applications of gelatin. In addition, different reviews have examined the potential use of plant-based hydrocolloids as gelling agents, mainly focusing on their physicochemical properties, extraction processes and broad application fields (e.g., pharmaceutical, biomedical or general food structuring) [8,9,10,11,12]. However, to the best of our knowledge, a review specifically centered on the replacement of animal gelatin in food gel applications is still missing. In particular, none of the most recent review articles explicitly focuses on the effective substitution of animal gelatin by plant-based hydrocolloids in real food products, nor on the technological implications of this substitution, but rather on their general structure–function relationships and broad application fields. In this work, we aim to fill this gap by providing an updated overview of the main hydrocolloids that can function as gelatin substitutes in food gels, either individually or in combination. Special emphasis is placed on κ-C and its synergistic mixtures, highlighting their potential, advantages and current challenges as next-generation gelling agents for food applications.

## 2. Animal Gelatin: The Traditional Standard for Gels

Animal gelatin (AG) has long been regarded as a cornerstone ingredient due to its versatility and unique functional properties. Universally recognized as a special hydrocolloid, it performs multiple roles across a wide range of applications in the food industry, as well as in pharmaceutical and biomedical fields. In foods, AG provides structure, texture, and sensory appeal, while in pharmaceuticals, it is widely used for capsules and microspheres, and in biomedicine, it serves wound dressings and tissue regeneration [9]. Derived from the partial hydrolysis of collagen from animal skin, connective tissue, and bones [4], gelatin owes its enduring success to a combination of features that are difficult to reproduce with alternative hydrocolloids.

The distinctive attributes of gelatin make it the benchmark against which other gelling agents are evaluated. An effective substitute should aim to replicate, at least partially, the following properties [13]:

- Melt-in-the-mouth perception: gelatin melts just below human body temperature, producing a characteristic sensory experience accompanied by rapid flavour and aroma release. This unique perception is particularly difficult to achieve with plant-based polymers.
- Thermally reversible gelation: gelatin gels are reversible, liquefying upon heating and re-forming upon cooling.
- Multifunctionality: a single biopolymer simultaneously provides gelling, thickening, water-binding, emulsifying, foaming, and film-forming capabilities.
- Customizability: commercial gelatin is available in a broad range of gel strengths and particle sizes, allowing fine-tuning for diverse industrial applications.
- Ease of use: gelatin readily gels within the natural pH range of most foods without requiring additional salts, sugars, or acids, unlike many plant-derived hydrocolloids.

This unique combination of sensory, functional, and technological advantages explains why gelatin has maintained its traditional role as the reference standard, despite the increasing demand for alternatives driven by ethical, religious, and lifestyle considerations, as well as by concerns over potential contamination risks [14].

## 3. Development of Gelatin Alternative from Polysaccharides

It is evident that the task of finding an ideal alternative to mammalian gelatin is very difficult, if not impossible. According to Karim et al. [15], the approach to develop gelatin alternatives for the food industry should follow an application/process-specific strategy, since it is unlikely that a single universal ingredient will be able to replace gelatin across all food systems.

Among these strategies, hydrocolloids represent the most promising choice [16]. They are a broad class of high-molecular-weight hydrophilic biopolymers—mainly polysaccharides and, to a lesser extent, proteins—characterized by their ability to disperse, swell, or dissolve in aqueous environments [17]. Derived from plants, seaweeds, and microorganisms, with additional semi-synthetic derivatives such as cellulose-based gums, hydrocolloids perform a wide spectrum of functions in food systems, ranging from thickening and stabilization to gelation [18]. Within this group, plant-based polysaccharides such as starch, pectin, alginate, carrageenan, arabic gum, locust bean gum, xanthan, konjac, and guar gum are particularly relevant [19]. Compared to animal-derived gelatin, the above-mentioned hydrocolloids offer significant advantages: they are plant-based, sustainable, and suitable for vegetarian and vegan diets. More importantly, their diverse gelation mechanisms not only make them promising candidates for replacing gelatin in conventional applications but also expand the possibilities for designing next-generation structured food gels [20].

### 3.1. Carrageenan: Physicochemical and Functional Properties

The general term refers to a family of high molecular weight polysaccharides composed of repeating units of 1,3-linked β-D galactopyranose and 1,4-linked 3,6-anhydro α-D-galactopyranose [21,22]. Different types of carrageenans are classified using Greek letters (κ, ι, λ, μ, θ, ν, etc.). Among them, κ-Carrageenan (κ-C), ι-Carrageenan (ι-C) and λ-Carrageenan (λ-C) are the most used ones. They differ in the number of sulphate groups: κ-C has one, ι-C has two, and λ-C has three. In addition to the overall sulfate content, the distribution of sulfate groups along the polysaccharide chain and the molecular weight strongly affect double-helix formation and gelation behavior. A lower degree and a more regular distribution of sulfation, as typically found in κ-C, promotes ordered double-helix aggregation and the formation of stronger, more rigid gels, whereas higher and more irregular sulfation, as in λ-C, hinders helix association and prevents gel formation.

At low concentrations, carrageenans produce a viscous solution that acts as a thickening and stabilizing agent. At higher concentrations, κ- and ι-carrageenan exhibit similar properties. In aqueous solution at elevated temperatures, they dissolve as random coils, whereas at lower temperatures they form a 3D polymeric network through sulphate groups and the 3,6-anhydro-D galactopyransyl ring. Typically, κ-carrageenan forms firm, brittle gels that may undergo syneresis (water exudation), whereas ι-carrageenan forms elastic, soft gels that usually resist syneresis.

Mixtures of carrageenan types have garnered attention due to the unique properties they offer for specific industrial applications, which are often unattainable with single-type carrageenan gels [23]. λ-carrageenan is unable to form a gel alone and has a random coil conformation at all temperatures. However, recent research showed that gelation of λ-carrageenan is possible in the presence of trivalent ions [24]. This finding broadens the potential applications of λ-carrageenan beyond its conventional use as a viscosity modifier.

Carrageenan solubility is strongly dependent on chemical structure. κ-C is less soluble due to its lower sulfate content and anhydrous residues, whereas ι-carrageenan exhibits higher solubility. Temperature is the main factor influencing dissolution, heating being the most effective method [25,26]. All carrageenan solutions are stable at pH above 6; between pH 3.5 and 6, gels remain stable once formed, while below pH 3.5, they are unsuitable as they undergo hydrolysis [27,28].

The global carrageenan market size was valued at USD 871.66 million in 2022 and is expected to expand at a Compound Annual Growth Rate (CAGR) of 5.4% from 2023 to 2030 [29,30]. This expansion is driven by increasing use in dairy and processed meat products, where carrageenan contributes to fatty mouthfeel and binds water, as well as by a growing preference for plant-based ingredients to replace animal-derived ones such as gelatin. κ-C represents the dominant market segment and is expected to grow at a CAGR of about 7%, with applications ranging from texture improvement in cottage cheese and viscosity control in dairy desserts to its role as a binder and stabilizer in meat products. Beyond food, κ-C is also used in cosmetics, for instance, to stabilize toothpaste and to enhance the sensory properties of lotions and shampoos.

The widespread use of κ-carrageenan is attributed to its unique physicochemical characteristics, which depend not only on chemical composition but also on the microstructure of its hydrogels [29]. Because of its superior gelling ability, κ-C is the focus of the present work, with its gelation mechanisms and properties discussed in the following, based on recent literature.

#### 3.1.1. Safety and Regulation of κ-Carrageenan-Carrageenan

Carrageenans have a long history of use in Asia and Europe and were first commercialized in the early 19th century as a powder product [29]. Today, they are food additives approved by the Food and Drug Administration (FDA) and Generally Recognized as Safe (GRAS). They are widely used as gelling, thickening, and emulsifying agents in food products, as pigment dispersion aids and drug encapsulation materials in cosmetics and pharmaceutical formulations, and as biomaterials for artificial tissue engineering in biomedical applications [30]. Carrageenan (E 407) and processed Eucheuma seaweeds (E 407a) are authorized food additives in the EU, with purity and safety criteria established under Regulations (EC) 1333/2008 and (EU) 231/2012. The temporary group Acceptable Daily Intake (ADI), i.e., the estimated amount that can be consumed daily over a lifetime without appreciable health risk, is 75 mg/kg body weight/day. Safety concerns focus on low molecular weight fractions (<50 kDa) and degraded carrageenan (poligeenan), which are not authorized due to observed adverse effects in animal studies, while high-molecular-weight carrageenan is generally considered safe. Exposure estimates indicate that high consumers, particularly young children, may exceed the temporary ADI. These additives are widely used in products such as flavored dairy, bakery, and beverages, and while generally regarded as safe, the presence of low molecular weight fractions and degradation products has contributed to scientific debate and cautious consumer perception. Specific restrictions, such as prohibiting E 407/E 407a in mini-cup jelly products due to choking risk [31].

#### 3.1.2. Gel Strength and Application

Gels formed by κ-C alone are often described as strong but brittle, prone to syneresis, and lacking the elasticity and cohesive mouthfeel associated with animal gelatin. Increasing κ-C concentration enhances gel strength, reflected in higher elastic moduli (G′) and greater durability. However, this improvement comes at the cost of higher sol–gel transition temperatures and reduced flexibility [32]. At concentrations above 4–5 wt%, κ-C hydrogels display stable and long-lasting structures, supported by extensive double-helix aggregation, whereas dilute gels (2–3 wt%) show poor durability and low fracture resistance [33,34,35]. Electron microscopy reveals that freeze-dried κ-C gels possess porous and fragile microstructures, consistent with their limited mechanical resilience [36].

**Figure 1:**
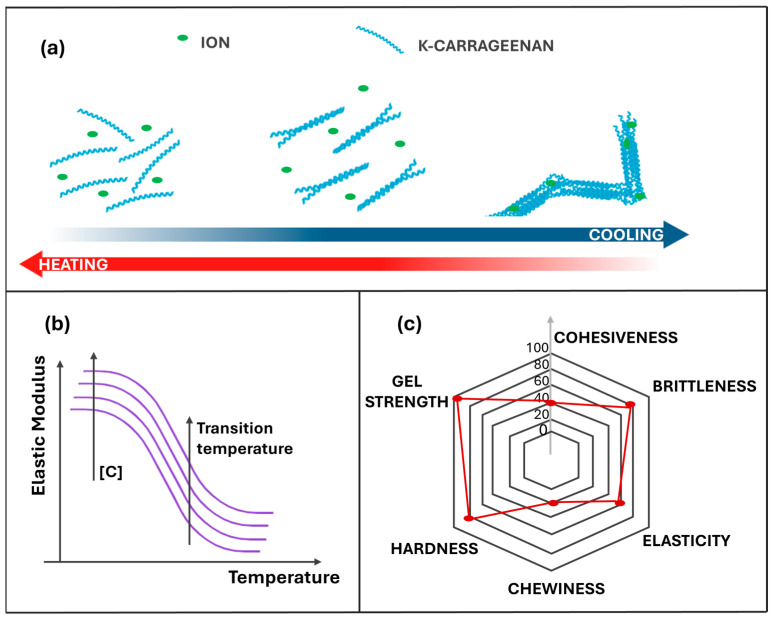
(**a**) Schematic representation of the κ-carrageenan gelation mechanism. Upon cooling, the random coil chains progressively form double helices stabilized by specific ions (mainly K^+^), which aggregate to create a three-dimensional network. (**b**) Conceptual Effect of κ-carrageenan concentration on the elastic modulus (G′): increasing polymer concentration shifts the sol–gel transition to higher temperatures and enhances gel strength. Axes, units, and values are illustrative. (**c**) Representative radar plot illustrating the typical textural attributes of κ-Carrageenan gels, highlighting their high gel strength and hardness, but limited elasticity and cohesiveness compared with gelatin-based systems.

Gel formation is strongly ion-dependent, with potassium ions particularly effective in stabilizing double-helix junctions and cross-linking adjacent helices [37]. Calcium and barium ions can also reinforce the network, albeit less selectively, while sodium ions generally yield more brittle gels. κ-C shows clear thermoreversibility, with sol–gel (Tsol–gel) and gel–sol (Tgel–sol) transition temperatures increasing linearly with concentration and potassium content. The gelation and melting temperatures were determined by rheological measurements as the G′-G″ cross-over point [38]. Tsol–gel values typically range from ~35 °C at low concentrations to nearly 60 °C at higher ones, well above the melting point of gelatin (~35 °C). This explains why κ-C gels lack the “melt-in-the-mouth” sensory quality of gelatin-based products [21]. Figure 1 schematically illustrates the κ-carrageenan gelation mechanism (panel a), the conceptual effect of concentration on the elastic modulus and sol–gel transition temperature (panel b), and the typical sensory attributes of the gel (panel c). Panels are illustrative and not based on experimental measurements. Taken together, these observations highlight κ-C as a plant-based hydrocolloid capable of forming thermoreversible gel systems whose mechanical performance and melting behavior are highly concentration-dependent. On the one hand, increasing κ-C content effectively enhances gel stiffness, bringing the elastic response closer to that of gelatin; on the other hand, such a reinforcement is systematically accompanied by a marked rise in brittleness and in the melting temperature. The latter is a critical parameter in many food applications. From a sensory perspective, this trade-off is reflected (as conceptually summarized in panel c) in a progressive increase in hardness, understood as the force required to fracture the gel, together with a concomitant decrease in chewiness and cohesiveness, indicative of a more fragile and less cohesive matrix. Thus, κ-C emerges as a realistic and promising alternative to animal gelatin. However, its use as a direct one-to-one replacement appears intrinsically limited, and optimal exploitation of its potential will likely require specific formulation strategies or combinations with other structuring agents.

### 3.2. κ-Carrageenan Gels in Complex Food Systems

The addition of sugars substantially modifies the mechanical and thermal performance of κ-C gels. Sucrose, glucose, fructose, and related polyols up to ~40% *w*/*w* enhance gel rigidity, elastic modulus, and thermal stability by strengthening junction zones through hydrogen bonding between sugar hydroxyl groups and sulfate or hydroxyl groups of κ-C [39,40]. Beyond this level, however, excessive sugar immobilizes water molecules essential for helix aggregation, weakening the gel. Rheological studies indicate that yield stress increases with sucrose addition, while yield strain is more sensitive to κ-C concentration [41]. Thermal analyses also confirm a systematic upward shift in melting and gelling temperatures with higher sugar levels, reflecting enhanced network stabilization. Nevertheless, excessive sugar disrupts molecular ordering and reduces transition enthalpy [42]. At high solids content, κ-C behaves differently from gelatin: while sugars promote gelatin self-association, κ-C gels tend to disaggregate. At very high solids concentrations (73–80%), both biopolymers may undergo vitrification, yielding glassy networks with extremely high moduli (≈10^10^ Pa). From a sensory perspective, sugar addition generally stiffens κ-C gels, although excessive levels can produce gummy textures, less pleasant than the smooth and elastic dissolution of gelatin [43].

Blends with ι-carrageenan (ι-C) are an effective strategy to mitigate brittleness and tune elasticity. While κ-C alone forms rigid, brittle networks stabilized by K^+^, ι-C produces softer and more elastic structures [44]. Their combination provides a wider range of textures, closer to the mechanical balance of gelatin. For example, mixtures at 0.15% κ-C and 0.85% ι-C yield soft, cohesive gels with minimal syneresis, suitable for desserts [45]. These improvements significantly expand the textural space of κ-C gels; however, the behaviour of κ-C/ι-C systems still remains distinct from that of gelatin, also due to their relatively high melting temperature, which prevents the characteristic ‘melt-in-the-mouth’ perception of animal gelatin gels.

Interactions with proteins, particularly milk proteins, have been widely exploited in dairy systems such as flans, puddings, chocolate milk, and processed cheese [46]. κ-C selectively interacts with κ-casein on the surface of casein micelles, forming electrostatic complexes that stabilize suspensions and promote weak gelation at concentrations as low as 0.01–0.02%. Such interactions prevent, for example, cocoa sedimentation in chocolate milk or serum separation in ice cream mixes. At higher concentrations (≥0.1%), κ-C self-association dominates, producing self-supporting gels whose rigidity depends strongly on the ionic environment [47]. Differential Scanning Calorimetry (DSC) shows that sodium caseinate has little effect on the helix–coil transition, but broadens melting peaks, indicating heterogeneous junctions and reduced thermal uniformity. From a sensory perspective, κ-C improves body and creaminess perception in skimmed milk and contributes firmness and elasticity to dairy gels, although excessive amounts can promote syneresis and lead to undesirable rigidity. Compared with gelatin, κ-C/protein systems generally provide higher firmness and improved water retention but lack the low melting point and cohesive resilience of animal gels [48].

Overall, κ-C offers a versatile platform for generating diverse textures and mechanical strengths, modulated by concentration, ions, sugars, and blending with other hydrocolloids or proteins. Although it cannot fully reproduce gelatin’s unique ‘melt-in-the-mouth’ perception, κ-C remains a key ingredient in vegan and dairy formulations, valued for its thermoreversibility, tunability, and cost-effectiveness.

## 4. Synergistic Combinations with Other Hydrocolloids

Despite its widespread use as a plant-based gelling agent, κ-C suffers from inherent limitations such as brittleness, low flexibility, and susceptibility to syneresis. A well-established strategy to overcome these drawbacks is blending with other hydrocolloids, which enhances structural stability, improves water retention, and broadens the range of achievable textures. Such synergistic systems enable the tailoring of gel properties for specific applications, providing functionalities unattainable with κ-C alone.

### 4.1. κ-Carrageenan and Locust Bean Gum

Locust bean gum (LBG, E401), or *carob gum*, is a galactomannan obtained by milling the endosperm of carob tree seeds into a white to creamy white powder [49]. It is a food additive approved by the Food and Drug Administration (FDA) and Generally Recognized as Safe (GRAS). Its structure consists of a linear β-D-mannose backbone (1,4-linked) carrying α-D-galactose side groups through 1,6-linkages [50]. The typical mannose/galactose (M:G) ratio is about 4:1, although it varies with processing [51]. Compared with other galactomannans—guar (M:G ≈ 2:1) or tara gum (M:G ≈ 3:1)—LBG contains less galactose, which reduces its intrinsic solubility The global LBG market was valued at about USD 269 million in 2023 and is projected to grow at over 2.4% (CAGR) between 2024 and 2032, reflecting its increasing use across multiple sectors [52]

**Figure 2:**
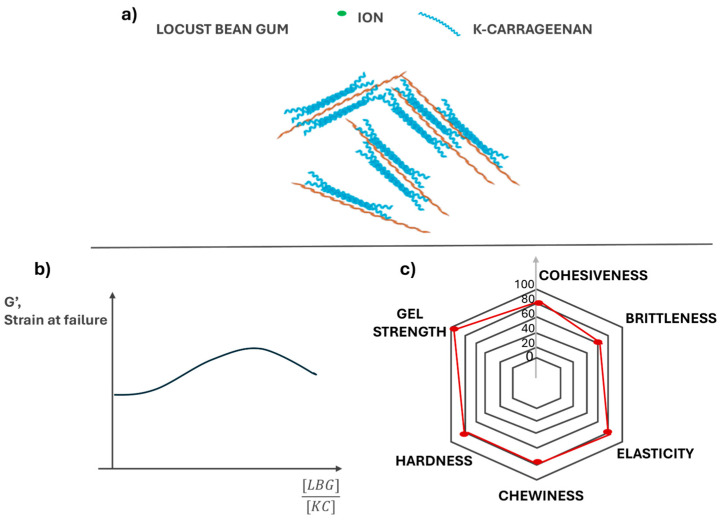
(**a**) Schematic representation of the synergistic interaction between κ-carrageenan (κ-C) and locust bean gum (LBG). (**b**) Effect of the κ-C/LBG ratio on the elastic modulus (G′): increasing the proportion of LBG enhances elasticity and network flexibility up to an optimal ratio. (**c**) Representative radar plot illustrating the typical textural attributes of κ-carrageenan gels.

Under normal conditions, LBG does not form a self-standing gel, yet gelation can be induced by freeze–thaw cycles, high sucrose concentrations, or co-gelling with other hydrocolloids such as κ-C [53]. Blends of κ-C and LBG produce gels with greater strength and improved functionality than either polymer alone [54]. κ-C solutions diluted below their individual gelling threshold can form gels upon LBG addition, providing clear evidence of synergistic interactions. The growing demand for plant-based gels has therefore stimulated extensive research into κ-C/LBG interactions across food, cosmetic, pharmaceutical, and packaging applications. Beyond food, LBG’s biodegradability, pH stability, and film-forming ability support its use as a thickener, stabilizer, and component of edible coatings or biodegradable films [55]. Figure 2 illustrates the synergistic behavior between κ-C and locust bean gum (LBG): panel (a) shows a possible interaction mechanism between κ-C and LBG; panel (b) reports the trend of the elastic modulus as a function of the κ-C/LBG ratio; and panel (c) presents a radar plot of the main sensory attributes.

At the molecular level, several models have been proposed to explain this synergy. One describes a coupled network in which unsubstituted mannan regions of LBG align with κ-C double helices at specific junction zones, stabilized by potassium ions [10]. Tako and Nakamura reported that synergy is observed primarily with the K^+^ form of κ-C, whereas sodium or calcium forms are ineffective. Cairns and co-workers [56] proposed instead that LBG persists as a dispersed phase within a continuous κ-C network, consistent with gelation–melting studies. NMR analyses by Rochas and co-workers [57] further indicate that carrageenan may promote mannan–mannan self-association, contributing to junction formation.

Complementary rheological, DSC and electron spin resonance (ESR) data support an interaction-driven reinforcement of the κ-C network. LBG addition shifts gelation to higher temperatures (≈53 °C vs. 40 °C) and markedly increases elasticity, indicating a denser and more cohesive matrix. Calorimetric profiles exhibit an additional high-temperature transition associated with κ-C ordering and κ-C/LBG interactions, while broader heating peaks suggest enhanced aggregation. ESR studies show that κ-C helices assemble at elevated temperatures, whereas LBG mobility remains largely unaffected, pointing to limited but cooperative binding. Taken together, these observations support a model in which κ-C helices locally cross-link short, relatively rigid mannan segments of LBG, reinforcing the three-dimensional network and yielding gels with improved strength and stability.

#### Gel Strength and Application

The mechanical and sensory behaviour of κ-C blended with LBG has been extensively characterised because LBG, although non-gelling on its own, aligns with κ-C helices to modulate network formation. Rheological studies [58] show that increasing LBG softens κ-C gels while preserving cohesion. At κ-C:LBG ratios around 7:3–6:4, fracture tests reveal maximum strain at failure and energy, indicating a ductile structure, whereas carrageenan-rich gels (≥9:1) remain brittle. Salt influences this balance: low levels (~0.25% KCl/NaCl) favor strength and elasticity, while higher concentrations (~1%) increase crosslinking, raising fracture stress but reducing strain. Mechanistically, the mannan backbone of LBG aligns with κ-C double helices during cooling, while potassium stabilizes junction zones, reinforcing the three-dimensional network [9].

Fernandes et al. [59] describe κ-C/LBG mixtures as one of the most widely exploited synergistic systems in food formulation. These blends gel at roughly half the total polymer concentration required for κ-C alone, as galactomannan chains promote helix formation and aggregation. The sol–gel transition is unusually sharp: slight changes in composition (e.g., 21:79 to 22:78) cause steep rises in apparent viscosity at low shear, reflecting rapid network development. Russo Spena et al. [60] provided an extensive rheological–textural survey of aqueous κ-C/LBG systems (κ-C 1–2% *w*/*w*; LBG/κ-C 1:2–1:10) benchmarked against 6.67% porcine gelatin. Texture profile analysis (TPA) indicates that κ-C/LBG blends achieve higher stress at break and improved water retention, but strain and cohesiveness remain inferior to gelatin (~130% strain, 94% springiness), highlighting a persistent gap in replicating gelatin’s characteristic deformability. Large-amplitude oscillatory shear reveals tan δ ≈ 0.1 versus gelatin’s tan δ ≈ 0.01, reflecting greater energy dissipation and a less elastic network. Microscopy confirms a continuous but coarser microstructure, consistent with sensory reports of firmer, less resilient mouthfeel. These observations underscore that, despite clear synergistic reinforcement, κ-C/LBG systems cannot fully mimic the smooth, “melt-in-the-mouth” perception of animal gelatin, limiting their performance in applications where gelatin’s textural subtlety is critical.

Additional synergy emerges in composite systems. Lu et al. [61] examined casein/κ-C/LBG ternary gels for processed cheese analogues, showing that modest LBG additions (0.1–0.3%) enhanced hardness, chewiness, storage moduli, and water-holding capacity, confirmed by DSC, temperature sweeps, and low-field NMR. Micrographs displayed a denser honeycomb-like microstructure; excessive LBG (>0.3–0.4%) caused phase separation and loss of strength. Murayama et al. (1995) [23] compared κ-C with galactomannans of differing galactose/mannose ratios, finding κ-C/LBG blends at 1:1 mass ratio produced the greatest hardness, fracture stress, and strain, outperforming κ-C alone and κ-C/tara gum, whereas guar gum failed to promote gelation. Sensory analysis rated κ-C/LBG gels as mechanically robust yet perceived as stiff, while κ-C/tara gum offered a more palatable balance.

Despite broad industrial adoption, aspects of κ-C/LBG synergy remain debated. The κ-C:LBG ratio yielding peak G′ spans 8–70% LBG depending on polymer and potassium levels [23]. Unlike pure κ-C, where carrageenan or K^+^ simultaneously raises gel strength and gelation temperature, κ-C/LBG systems often show a pronounced G′ peak but a monotonic decrease in gelling temperature with rising LBG content. These patterns underscore that reinforcement arises from specific coil–helix interactions rather than solely from ion-mediated crosslinking.

Overall, κ-C/LBG mixtures convert the inherently brittle κ-C gel into a softer yet cohesive matrix with tunable strength, viscoelasticity, and improved water retention. Balanced formulations reduce syneresis and broaden the sensory profile, enabling applications in vegan confectionery, dairy analogues, and other plant-based gels. Nonetheless, elevated gel–sol transition temperatures and lower elasticity relative to gelatin remain significant hurdles for fully replicating the mechanical–sensory signature of animal gels.

### 4.2. κ-Carrageenan and Konjac Glucomannan (KGM)

Konjac glucomannan (KGM) is a high-molecular-weight polysaccharide extracted from the tubers of *Amorphophallus konjac* and valued as a functional food ingredient [8]. As a food additive by the US FDA, its use in the EU is limited to less than 1% content, alone or with other thickeners. KGM is prized for its technological versatility, health benefits—including appetite regulation, gut microbiota modulation, cholesterol reduction, and support in type-2 diabetes—and biodegradability, which also supports pharmaceutical applications.

Chemically, KGM consists of β-(1→4)-linked D-mannose and D-glucose units (molar ratio of approximately 1:1 to 1.6:1) with an average molecular weight around 10^6^ Da (commercial samples 2 × 10^5^–2 × 10^6^ Da) [62]. Its strong hydration and solubility allow for the formation of viscous solutions at low concentrations, retaining up to 50 times its weight in water. Its physicochemical behavior is modulated by pH, ionic environment and processing: (i) under alkaline conditions, removal of acetyl groups promotes hydrogen bonding, leading to partial crystallization and the formation of thermally irreversible gels; (ii) in neutral aqueous solutions, KGM primarily acts as a thickener, imparting high viscosity; (iii) when blended with other hydrocolloids—such as carrageenan, xanthan or locust bean gum—thermally reversible hydrogels can be obtained [63]. The Konjac market is expected to grow with a CAGR of 7.1%.

Synergistic interactions arise when KGM is blended with κ-C. κ-C/KGM gels exhibit stronger associative interactions and enhanced mechanical properties than κ-C/LBG blends [64]. Early studies suggest that KGM reinforces κ-C networks via adsorption onto carrageenan helices, with mainly physical interactions, while a dual-junction model proposes primary κ-C junctions and secondary, weaker KGM-mediated junctions, enhancing cohesion and elasticity [64].

Recent micro-DSC, rheometry, and AFM studies show that KGM promotes the coil-to-helix transition of κ-C, stabilizes helices via adsorption, and fosters the formation of a dense, ordered three-dimensional network, yielding stronger and more homogeneous gels than κ-C alone [65].

#### 4.2.1. Safety and Regulation of Konjac Glucomannan

Konjac gum (E 425 i) and konjac glucomannan (E 425 ii) are authorized food additives in the EU under Regulation (EC) 1333/2008. Based on refined exposure assessments at current use levels (maximum permitted level, MPL, 10 g/kg), no safety concern has been identified for the general population, and a numerical ADI was not considered necessary. Both konjac gum and glucomannan are of low acute toxicity, are not absorbed intact in the gastrointestinal tract, and are largely fermented by the colonic microbiota, yielding short-chain fatty acids without safety concerns. E 425 is widely used across several food categories, including flavoured dairy, beverages, and bakery products, and is classified as a food additive by the US FDA. In the EU, its use is generally limited to concentrations below 1%, either alone or in combination with other thickeners. Safety restrictions mainly relate to physical hazards associated with its strong swelling and gel-forming properties, which have led to bans on mini-cup jellies. At the same time, konjac glucomannan has attracted considerable interest due to its technological versatility and reported health benefits, such as appetite regulation, modulation of gut microbiota, cholesterol reduction, and support in the management of type-2 diabetes and insulin resistance. Its biodegradability further enhances its appeal for pharmaceutical and drug-delivery applications [8].

#### 4.2.2. Gel Strength and Application

The pairing of κ-C with KGM has garnered significant attention due to its ability to rectify the inherent shortcomings of each gel former when used alone. Compared to the brittle, thermoreversible κ-C gels, KGM is highly viscous and water-binding but does not gel under standard conditions. Their combination synergistically enhances gel strength, elasticity, thermal stability, and sensory appeal.

Rheological studies show that optimal κ-C:KGM ratios (~1:7 *w*/*w*, KGM:κ-C) maximize gel firmness and viscoelastic moduli [66]. Excess KGM increases strain at failure and stress but lowers overall modulus, highlighting a trade-off between rigidity and extensibility. Dynamic oscillatory tests confirm that KGM reinforces the network, maintaining G′ ≫ G″ over a broader frequency range and increasing fracture resistance [67].

Microstructural analyses reveal that KGM promotes κ-C chain helix formation, which aggregates into trigonal motifs forming a dense 3D network. Tethering between glucan and carrageenan segments generates nanoscale domains that expand with KGM content, correlating with enhanced mechanical strength. An alternative model describes a “nanogel junction network,” where KGM chains are crosslinked via κ-C nanoclusters, producing high tensile resistance and controlled swelling [66].

Thermal studies support these structural insights. DSC often shows dual melting peaks, with the lower one corresponding to κ-C/KGM junction dissociation and the higher one to κ-C helix melting. Hydrolyzed KGM raises the gel–sol transition temperature and shifts G′/G″ crossover to higher ranges, indicating stabilized junctions. Additional additives, such as maltodextrins or cyclodextrins, can further tune melting, recrystallization, and hysteresis behavior [68].

Functionally and sensorily, κ-C/KGM gels transform brittle, collapse-prone matrices into coherent, mouth-pleasing gels with improved elasticity, chewiness, and water retention, reminiscent of gelatin. Applications include milk puddings, vegan confections, meat analogues, gelled beverages, and 3D-printing inks, exploiting rapid sol–gel transitions and reinforced network formation for precise deposition [69].

In summary, κ-C/KGM mixtures harness synergistic interplay between carrageenan helices and glucomannan chains to produce gels with a rare combination of strength, elasticity, thermal robustness, and sensory compatibility. Ongoing research continues to deepen our mechanistic understanding and expand their technological versatility across the spectrum of plant-based functional foods and nutraceuticals [37].

## 5. Other Potential Polysaccharide-Based Gelatin Alternatives

Beyond κ-carrageenan systems, several other hydrocolloids have been investigated—though often less systematically—as potential gelatin replacers. These approaches remain comparatively underexplored but offer promising, and sometimes highly application-specific alternatives.

Xanthan, the extracellular polysaccharide produced by *Xanthomonas campestris*, does not form gels on its own, yet it can generate strong, cohesive networks when combined with specific plant polysaccharides, particularly LBG and KGM [10]. Agoub et al. [12] showed that mixtures of pyruvate-free xanthan and KGM can mimic the softening of gelatin-based gels at mouth temperature in moderately acidic systems (pH 3.5–4.0), such as fruit jellies. Although the textural match is not exact, the marked decrease in modulus upon heating to body temperature suggests a realistic route to plant-based “melt-in-the-mouth” gels in acidic products.

Pectin-based systems provide another example where functionality is promising but inherently constrained. High-methoxyl (HM) pectins are generally poor direct substitutes for gelatin because they form thermally irreversible gels and require low pH and high soluble solids. Low-methoxyl (LM) pectins offer more flexible gelation conditions, yet at high sucrose levels, they also tend to pre-gel. In practice, blends of HM and LM pectins are used to tune texture: by adjusting their ratio together with Ca^2+^ concentration, sugar content, pH and degree of esterification, a broad palette of rheological behaviours can be achieved [70]. Still, the need for tight control of formulation variables and the lack of a sharp, reversible melting transition limit the extent to which pectin gels can replicate the sensory profile of gelatin.

Within this framework, numerous authors proposed pectin as a gelatin replacer in marshmallows [70,71]. While pectin-based aerated gels do not fully reproduce the strong, elastic structure of gelatin, the non-animal origin, favourable flavour release and good heat stability make selected HM pectins at low pH an attractive option. Overall, among the hydrocolloids tested, only pectin, carrageenan, or their combinations appear capable of approaching gelatin-like textures, though they still fall short of matching its precise melt-in-the-mouth behaviour.

Starch- and fibre-based systems represent a more “composite” strategy. Wang et al. [72] reported that a dual-modified starch combined with a wheat fibre gel could replace gelatin in yoghurt when used at an optimal 60:40 starch/fibre ratio. The starch fraction contributed smoothness and enhanced water-holding capacity, while the composite network improved stability at storage temperatures above 20 °C and reduced syneresis. Sensory tests showed no significant differences from gelatin-containing yoghurts, but these systems remain formulation-specific and do not readily generalize to more delicate, melt-sensitive gels.

High-acyl (HA) gellan gum has likewise been proposed as a versatile structuring agent with potential to emulate gelatin [15]. Gellan can deliver textures ranging from soft and elastic to firm and brittle, and the levels of glycerate and acetate substituents can be independently tuned. Blends of HA and low-acyl (LA) gellan produce intermediate textures, and partially deacylated HA gellan can closely approximate the texture of water-based dessert gels at ≈15% total solids, while offering a higher melt-set temperature beneficial for rapid setting and hot-climate stability. In high-solid systems, partially deacylated gellan more closely matches gelatin in brittleness, elasticity and cohesiveness than simple HA/LA blends. Nevertheless, as with other hydrocolloids, the melting behaviour does not fully reproduce the narrow thermal window and cohesive breakdown typical of gelatin.

Taken together, these xanthan-, pectin-, starch/fibre- and gellan-based systems illustrate that a range of plant-derived hydrocolloids can approach specific aspects of gelatin functionality—mechanical strength, water retention, or thermal stability—but typically only within restricted formulation windows and without fully capturing gelatin’s characteristic, reversible melt-in-the-mouth perception [73,74].

An overall comparison of the systems discussed in this review is provided in Table 1, which summarizes their key formulation variables, structure–property relationships and suitability as gelatin replacers.

## 6. Conclusions and Future Perspectives

Gelatin remains one of the most versatile hydrocolloids used in food, pharmaceutical and non-food applications. However, concerns related to its animal origin—tied to religious constraints, lifestyle choices, and potential risks of viral or prion transmission—have accelerated the search for alternatives. Among plant-derived candidates, κ-C, alone or in combination with other polysaccharides, has emerged as a particularly promising option. κ-C-based gels can deliver robust, thermoreversible networks, and their performance can be substantially improved by blending with locust bean gum (LBG), konjac glucomannan (KGM), starches and other hydrocolloids, thereby enhancing elasticity, water retention and the diversity of achievable textures.

Yet these systems still face intrinsic limitations, including the brittleness and syneresis of κ-C gels, the non-gelling nature of LBG and KGM in isolation, the dependence on elevated polymer and salt levels, and relatively high gel–sol transition temperatures compared with mammalian gelatin. Synergistic formulations can narrow the functional gap, but they typically require narrow processing windows (pH, ionic strength, temperature) and may involve trade-offs in sensory quality, ease of processing or economic feasibility. In fact, no single system can currently be regarded as definitively more promising than the others: different combinations and formulations tend to emulate specific aspects of gelatin functionality—such as firmness, water-holding capacity or melt-in-the-mouth behaviour—but none are yet able to fully replace animal gelatin. This inability to reproduce all attributes simultaneously ultimately underlines the uniqueness of mammalian gelatin.

Future work should therefore adopt a multi-pronged strategy. On the molecular side, enzymatic tailoring (controlled hydrolysis, oxidative coupling, enzyme-mediated cross-linking) and mild chemical modification (for instance, using food-grade polyphenols) could be exploited to fine-tune gel strength, elasticity and syneresis while preserving safety and clean-label expectations. Physical approaches—including high-pressure processing, irradiation, and 3D-printing-induced structuring—also warrant systematic exploration as routes to novel microstructures and improved thermal reversibility. In parallel, composite systems combining polysaccharides with proteins, peptides or nanofillers offer a powerful toolkit to broaden both mechanical performance and sensory response.

A deeper mechanistic understanding of synergistic interactions—coil–helix transitions, hydrogen-bonded junctions, nanogel junction networks—using advanced techniques such as NMR, AFM and molecular dynamics simulations will be essential for predictive formulation and industrial scale-up. Equally important are comprehensive rheological–sensory correlations and consumer studies, particularly in confectionery, dairy analogues and 3D-printed foods, together with life-cycle and sustainability assessments to validate the environmental benefits of plant-based systems.

Looking ahead, the next generation of gelatin alternatives will likely arise from the integration of advanced hydrocolloid blending, targeted molecular and physical modification, and, in parallel, biotechnological routes such as recombinant collagen and gelatin production. The successful development of cost-effective, sustainable and consumer-acceptable systems that approach—or selectively surpass—certain functional and sensory attributes of animal gelatin would not only broaden the technological options available to industry, but also respond to global demands for ethical, safe and environmentally responsible ingredients, while acknowledging that the complete functional equivalence of mammalian gelatin remains, for now, out of reach.

## Figures and Tables

**Table 1:** Summary of κ-carrageenan-based and other plant-derived hydrocolloid systems proposed as gelatin alternatives: key formulation variables, structure–property relationships, and main applications.

System/Hydrocolloid Combination	Typical Composition & Processing Conditions	Key Structural/Rheological Features	Sensory/Textural Attributes vs. Gelatin	Main Advantages/Limitations	Representative Food Applications	Key References
**κ-C**	κ-C ≈ 2–5 wt%; K^+^/Na^+^ as counterions; pH ≈ 6–7; heating above coil–helix transition and cooling under quiescent conditions	Strong, ion-dependent, thermo-reversible gels; G′ increases with κ-C and K^+^ concentration; porous, brittle network; high $T_{sol-gel}$ and $T_{gel-sol}$	Higher firmness than 6.67% gelatin; brittle fracture, limited deformability; lacks melt-in-the-mouth behaviour due to high melting temperature	**+** Strong gels at relatively low polymer level; good water binding and stability. **−** Pronounced brittleness and syneresis; high melting temperature; limited elastic recovery	Water-based dessert gels, milk desserts, confectionery inclusions, 3D-printing of simple shapes	[20,29,30,31,32,40,52]
**κ-C/LBG**	κ-C ≈ 1–2 wt%; LBG/κ-C ≈ 1:2–1:10; low–moderate KCl/NaCl (≈0.25–1%); heating–cooling under quiescent conditions	Synergistic gelation: LBG promotes κ-C helix formation and aggregation; higher G′ at lower κ-C level; sharper sol–gel transition; denser network; reduced syneresis	Higher fracture stress and water retention than κ-C alone; more ductile behaviour at κ-C:LBG ≈ 7:3–6:4; however, strain and cohesiveness remain lower than gelatin; melt-in-mouth still not reproduced ($T_{gel-sol}$ ≈ 43–70 °C)	**+** Synergy allows reduction in κ-C; improved ductility, reduced syneresis, better water holding; widely used, label-friendly system. **−** Narrow optimal composition and salt window; thermal behaviour still far from gelatin; incomplete match of elasticity and resilience	Confectionery-type dessert gels, dairy and dairy analogues, thickened creams, 3D-printable gels and structured foods	[46,52,54,55,56,57,59]
**κ-C/KGM**	κ-C typically ≤ 1–1.5 wt%; KGM added at comparable or slightly higher level; pH ≈ neutral; heating–cooling; possible deacetylation of KGM for thermally irreversible gels	Stronger synergy than κ-C/LBG: KGM promotes κ-C coil–helix transition, stabilises helices by adsorption, and yields thick helical bundles; dense, homogeneous network; higher G′ and improved fracture properties at lower κ-C	Elastic, cohesive gels with high water retention; more homogeneous texture than κ-C alone; melt-in-mouth behaviour improved but still limited by relatively high melting temperature	**+** High water binding and viscosity; strong reinforcement of κ-C network; possibility of tailoring reversibility via KGM acetylation. **−** Process-sensitive (pH, ionic strength, KGM deacetylation); regulatory and consumer perception aspects for konjac; still not fully equivalent to gelatin	Milk puddings and dairy analogues, high-fibre gels, structured soft foods, potential 3D-printed systems	[60,61,62,63,64,65,67,69,70,71,72]
**κ-C/starches, fibres, other polysaccharide (xanthan, pectin, etc…)**	κ-C combined with starch, dietary fibres, xanthan or pectin; often high-solid systems (sugar, acids, flavours); thermal processing typical of confectionery/desserts	Mixed networks or phase-separated structures; κ-C provides gel backbone; starch/fibres contribute viscosity, opacity, and water binding; microstructure and viscoelasticity strongly formulation-dependent	Can approach gelatin-like firmness and chewiness in high-solid matrices; mouthfeel often less elastic and more pasty or short; melting behaviour broader and less sharp than gelatin	**+** Wide formulation flexibility; possibility to tailor texture through multi-component design; improved nutritional profile (fibres). **−** Complex, application-specific optimisation; thermal behaviour and breakdown in mouth rarely match gelatin	Jelly candies, fruit gels, reduced-sugar confectionery, bakery fillings, hybrid dairy analogues	[30,32,36,37,38,39,71]
**Gellan gum (HA, LA and blends)**	HA, LA or partially deacylated gellan; total solids ≈ 10–20%; cations (Ca^2+^, Na^+^, etc.); heating and controlled cooling	Gel texture tunable from soft/elastic (HA) to firm/brittle (LA); blends or partial deacylation give intermediate properties; high set and melt temperatures; strong, transparent gels	In suitable formulations, partially deacylated gellan can approximate gelatin-like brittleness, elasticity and cohesiveness in water-based dessert gels; however, melting window remains broader and at higher temperature than gelatin	**+** Very versatile, robust gels; good thermal stability and rapid setting; suitable for hot climates. **−** Sensory breakdown and melt-in-mouth perception still differ from gelatin; ion-sensitive; sometimes perceived as too brittle	Water gels and desserts, beverages, confectionery, structured sauces, plant-based dairy analogues	[9,12,17]
**Other plant-based hydrocolloids (xanthan-galactomannan, pectin, mixed systems)**	Various combinations (xanthan/LBG, HM or LM pectin, fibres, proteins); pH, sugar and Ca^2+^ content tuned to application	From weak, spreadable gels to firm elastic networks; cold-set or heat-set depending on system; strong control of syneresis and water activity in high-solid products	Often able to mimic specific aspects of gelatin (e.g., spreadability, cuttability, chewiness) in narrow formulation windows; thermal reversibility and narrow melting range usually not matched	**+** Clean-label, often high-fibre; good control of water activity and stability; adaptable to many matrices. **−** Typically case-by-case optimisation; rarely able to reproduce full sensory profile of gelatin	Jams, fruit preparations, bakery fillings, meat and dairy analogues, high-fibre desserts	[14,15,16,21]
**κ-C based systems for 3D printed foods**	κ-C alone or in synergy (LBG, KGM, starch, fibres); concentration and temperature selected to ensure yield stress and shape fidelity; extrusion-based printing	Clear yield stress and shear-thinning behaviour; rapid structure recovery after extrusion; thermo-reversible network enables post-printing setting; microstructure tunable with cooling rate and composition	Texture post-printing ranges from soft/creamy to firm gels; mechanical properties can approach gelatin-based references, though cohesiveness and melt-in-mouth are still lower	**+** Good printability and shape retention; compatibility with flavours, colours and active ingredients; potential for personalised nutrition. **−** Narrow processing window (temperature, concentration); long-term stability and sensory attributes still under development	3D-printed snacks, desserts, personalised gels for children/elderly, prototype plant-based confectionery	[29,40,71]

## Data Availability

No new experimental data were generated. All information presented is derived from published sources.
